# Case report: Looking for relationship between type 1 diabetes and chronic recurrent osteomyelitis: short literature review and case presentation

**DOI:** 10.3389/fendo.2024.1505382

**Published:** 2025-01-08

**Authors:** Ausra Snipaitiene, Laura Radzeviciute, Kristina Aleknaviciene, Rimante Dobrovolskiene, Ingrida Stankute

**Affiliations:** ^1^ Department of Pediatrics, Medical Academy, Lithuanian University of Health Sciences, Kaunas, Lithuania; ^2^ Medical Academy, Lithuanian University of Health Sciences, Kaunas, Lithuania; ^3^ Department of Genetics and Molecular Medicine, Medical Academy, Lithuanian University of Health Sciences, Kaunas, Lithuania; ^4^ Department of Endocrinology, Medical Academy, Lithuanian University of Health Sciences, Kaunas, Lithuania; ^5^ Institute of Endocrinology, Medical Academy, Lithuanian University of Health Sciences, Kaunas, Lithuania

**Keywords:** chronic recurrent multifocal osteomyelitis, type 1 diabetes, celiac disease, autoimmunity, autoinflammation

## Abstract

**Background:**

Childhood autoimmune disorders involve the immune system attacking its own tissues, leading to varied symptoms, while autoinflammatory disorders result from innate immune system dysregulation, both requiring extensive diagnosis and multidisciplinary management due to their complexity.

**Case presentation:**

We present a unique clinical case of a teenager with a combination of autoimmune and autoinflammatory disorders. The initial manifestation of hip pain, coupled with progressive symptoms over several years and findings in multiple magnetic resonance imaging (MRI) scans, culminated in the diagnosis of chronic recurrent multifocal osteomyelitis (CRMO). Subsequently, the patient was diagnosed with type 1 diabetes (T1D), celiac disease, and juvenile idiopathic arthritis.

The therapeutic course proved challenging, marked by unsuccessful attempts with nonsteroidal anti-inflammatory drugs (NSAIDs), and biphosphonates. However, a stable clinical status was ultimately achieved upon the introduction of methotrexate, concomitant with insulin therapy for diabetes and the implementation of a gluten-free diet for celiac disease.

**Conclusions:**

Our case showed that the combination of autoimmune and autoinflammatory diseases, brought not only a challenging diagnostic process, but also complicated treatment.

## Introduction

Autoimmune disorders in childhood encompass a diverse group of conditions where the immune system, which is designed to protect the body, mistakenly targets and attacks its own tissues. These disorders can affect various organs and systems, leading to a range of symptoms. The most common autoimmune disorders in childhood include: juvenile idiopathic arthritis (JIA), type 1 diabetes (T1D), coeliac disease, systemic lupus erythematosus, autoimmune thyroid disorders, inflammatory bowel disease, psoriasis, etc. (1, 2).

On the other hand, autoinflammatory disorders in childhood are a group of rare conditions characterized by recurrent episodes of systemic inflammation that are not driven by the immune system’s typical mechanisms of antigen recognition, as seen in autoimmune disorders. These disorders result from dysregulation of the innate immune system, leading to excessive inflammation and symptoms such as fever, joint pain, skin rashes, and inflammation of various organs. Some notable autoinflammatory disorders in childhood include: familial mediterranean fever, tumor necrosis factor receptor-associated periodic syndrome, cryopyrin-associated periodic syndrome, hyperimmunoglobulin D syndrome, chronic nonbacterial osteomyelitis, etc. (3).

Finding potential causes of inflammation and diagnosing autoimmune and autoinflammatory disorders in children often involves extensive examinations, imaging studies, and sometimes biopsy or genetic testing. Treatment strategies vary depending on the specific disorder and may involve medications to modulate the immune response, manage symptoms, and sometimes lifestyle modifications such as dietary changes. Given the complexity of these disorders, their management often involves a multidisciplinary approach. Collaboration between different pediatric specialists is crucial for effective management, although, it is sometimes challenging to achieve good clinical outcomes.

## Case presentation

In February 2017, an 11-year-old female of Caucasian ethnic background was referred to a pediatric rheumatologist due to persistent right hip pain, radiating to the knee and foot. Additionally, she reported painful leg movements accompanied by intermittent leg stiffness. Her medical history revealed no serious injuries, chronic illnesses, or surgeries. The patient had normal body composition. There were no instances of consanguinity or chronic diseases in her family history.

Upon physical examination, she was afebrile with a generally good overall condition, albeit displaying a slight limp. Painful right hip adduction and rotation in all directions, limited range of right hip joint movements, along with tenderness at sacroiliac joints projections, were noted. No pathological findings were observed in any other joints. The assessment of her skin and neurological status was normal.

Haematological parameters, including complete blood count (CBC) and C-reactive protein (CRP) level, were within normal limits. However, a slightly elevated erythrocyte sedimentation rate (ESR) of 14 mm/h (normal range: 0-11 mm/h) was identified. Furthermore, the patient exhibited low blood calcium and vitamin D levels. Initial radiological investigations, comprising X-rays and ultrasounds of the spine, pelvis, and lower extremities, revealed no noticeable pathology. Based on these findings, she was prescribed vitamin D, calcium supplements and nonsteroidal anti-inflammatory drugs (NSAIDs) as needed.

**Figure 1:**
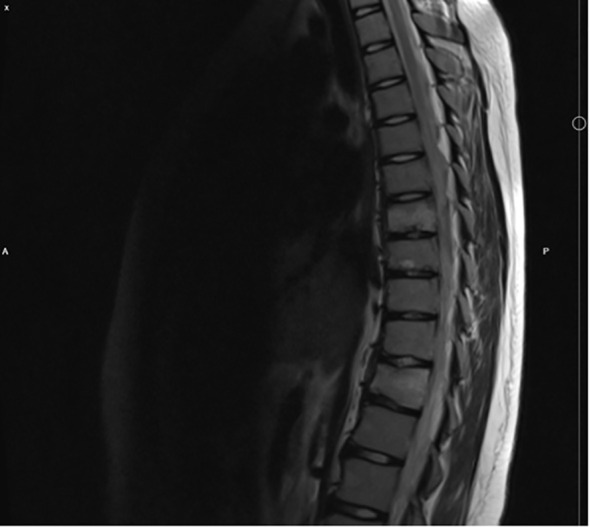
MRI of the spine, showing compression fractures of the Th7, Th8 and Th11.

During the next two years patient started feeling thoracic and lumbar pain each day, despite frequent use of NSAIDs. The physical examination revealed painful paravertebral palpation of the thoracic, lumbar, and sacral areas. On spine computed tomography (CT) there were signs of old compression fractures of the Th7, Th8, Th11 vertebrae and juvenile osteochondrosis. Subsequently, magnetic resonance imaging (MRI) of the spine revealed the same compression fractures in thorasic region with perifocal bone marrow edema, and additional stress fractures in the S2-S5 vertebrae (**Figure 1**). However, no radiological evidence suggestive of tumor presence was observed. Bone density evaluated by Dual Energy X-ray Absorptiometry (DXA), was normal according to reference values for gender and age, thus, osteopenia and osteoporosis were excluded. As well as, lung and skeletal tuberculosis (TB) was ruled out with negative serology.

For differential rheumatological diagnosis the patient underwent a large panel of immunological tests, presented in **Table 1**. However, despite a slightly elevated ESR, all tests (CBC, hepatic enzymes, kidney function, lactate dehydrogenase), were within normal ranges.

**Table 1:** Immunological examinations.

Test	Result
ANA	Negative
ANCA	Negative
RF	Negative
Serum protein electrophoresis	Normal
IgG with subclasses (IgG1, IgG2, IgG3)	Normal

ANA, antinuclear antibodies; ANCA, antineutrophil cytoplasmic antibodies; RF, rheumatoid factor; IgG, immunoglobulin G.

**Figure 2:**
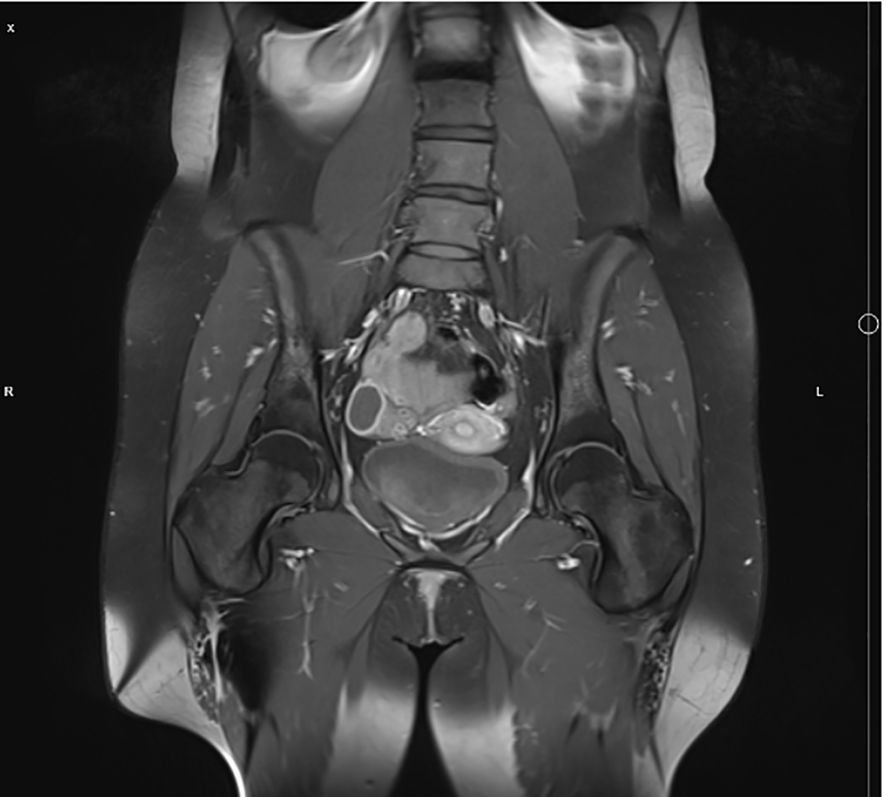
Pelvic MRI showing signs of bone marrow edema in both femoral heads.

Because of lumbar pain it was decided to perform pelvic MRI. It revealed lesions with bone marrow edema in both femoral heads (**Figure 2**). After evaluating all the data and radiological images, the multidisciplinary team agreed that patient’s signs and symptoms were characteristic for CRMO.

Given these findings, treatment with NSAIDs was prescribed and a proactive follow-up involved pelvic and spine MRI after six months. Despite sustained NSAIDs use, the patient presented with increased back pain and severe pain in her right knee six months later. An MRI of the right knee unveiled a tibial metaphyseal lytic lesion accompanied by bone marrow edema, while the spine MRI revealed additional sites of bone marrow edema. Considering the entire medical history and scientific literature, in June 2020 bisphosphonate therapy (pamidronate at 1 mg/kg per month) was initiated. Three doses were administered at monthly intervals, which resulted in significant clinical improvement and resolution of edema signs in femoral heads in repeated MRI.

However, by December 2020, the patient’s back and hip pain intensified, and a new lesion in the ischial bone was identified in MRI. Consequently, a second course of pamidronate at 1 mg/kg (three doses, one month apart) was deemed necessary. Just before the 4^th^ dose of pamidronate, a urine test revealed glucosuria, and a blood test indicated hyperglycemia (14.13 mmol/l), without exhibiting signs or symptoms characteristic of diabetic ketoacidosis. Consequently, the patient was screened for pancreatic antibodies, they confirmed diagnosis of T1D (Glutamic acid decarboxylase (GAD) autoantibodies 611.73 kU/l, highly elevated; insulin autoantibodies – negative; tyrosine phosphatase-related islet antigen 2 (IA-2) antibodies – negative). Glycosylated haemoglobin (HbA1c) was 11.6% (103 mmol/mol), prompting initiation of multiple daily insulin injections and diabetes education.

Additionally, thyroid function and celiac disease were screened according to the International Society for Pediatric and Adolescent Diabetes (ISPAD) guidelines. Tests revealed normal thyroid function with negative thyroid antibodies, however, tissue transglutaminase imunoglobulin (Ig) A level was elevated at 200 U/ml. Subsequent consultation with a pediatric gastroenterologist and screening for IgA and IgG class anti-endomysial antibodies and antibodies against deaminated gliadin (GAF3X) confirmed definite celiac disease, prompting recommendations for a gluten-free diet.

Till May of 2021 the patient was treated with maximum doses of NSAIDs (mainly diclofenac), however, follow-up MRI showed remaining lessions in the sacrum region. Reevaluation of the patients condition and imaging studies dynamics, biopsy of the sacrum lession was done. Hystological examination showed only sclerotic bone changes, no signs of malignancy including Langerhans hystiocytosis were seen.

Taking into account the worsening clinical symptoms, previous improvement after biphosphonate therapy, and disrupted pamidronate supply, due to the Covid-19 pandemic, it was decided to initiate treatment with zolendronic acid (ZA) in May 2022. However, if the 1^st^ dose of ZA seemed promising, the 2^nd^ dose brought no clinical improvement. Therefore, it was decided to start with methotrexate (MTX) 20 mg subcutaneously once per week in November 2022.

In addition, after consultation with genetician, whole exome sequencing (WES) and primary and secondary bioinformatics analyses were carried out by CeGaT GmbH, using their proprietary exome design to cover coding and disease-relevant regions. The Illumina NovaSeq6000 system was employed to sequence enriched regions, with an average coverage of 110x, and reads were processed and aligned to the hg19-cegat reference genome. Tertiary analysis, focused on skeletal and connective tissue disorders, was conducted, however, no pathogenic or likely pathogenic variants related to the clinical situation were found.

Up to the age of 18 (July 2024) the clinical symptoms were stable, dynamics in MRI showed at least some improvements, and no additional lesions, the pain was not worsening. Therefore, it was decided to continue with MTX, as recommended by Childhood Arthritis and Rheumatology Research Alliance (CARRA) guidelines. Her glycemic control has been consistently well-managed, with HbA1c levels typically under 7% and minimal glycemic fluctuations. She has used insulin pump therapy with the Medtronic 780 and ultra-fast-acting insulin (Fiasp). Adhering strictly to a celiac disease diet, she reported no gastrointestinal or related symptoms. At the age of 18 she was successfully transitioned to adult care, provided mainly by rheumatologist and endocrinologist.

**Figure 3:**
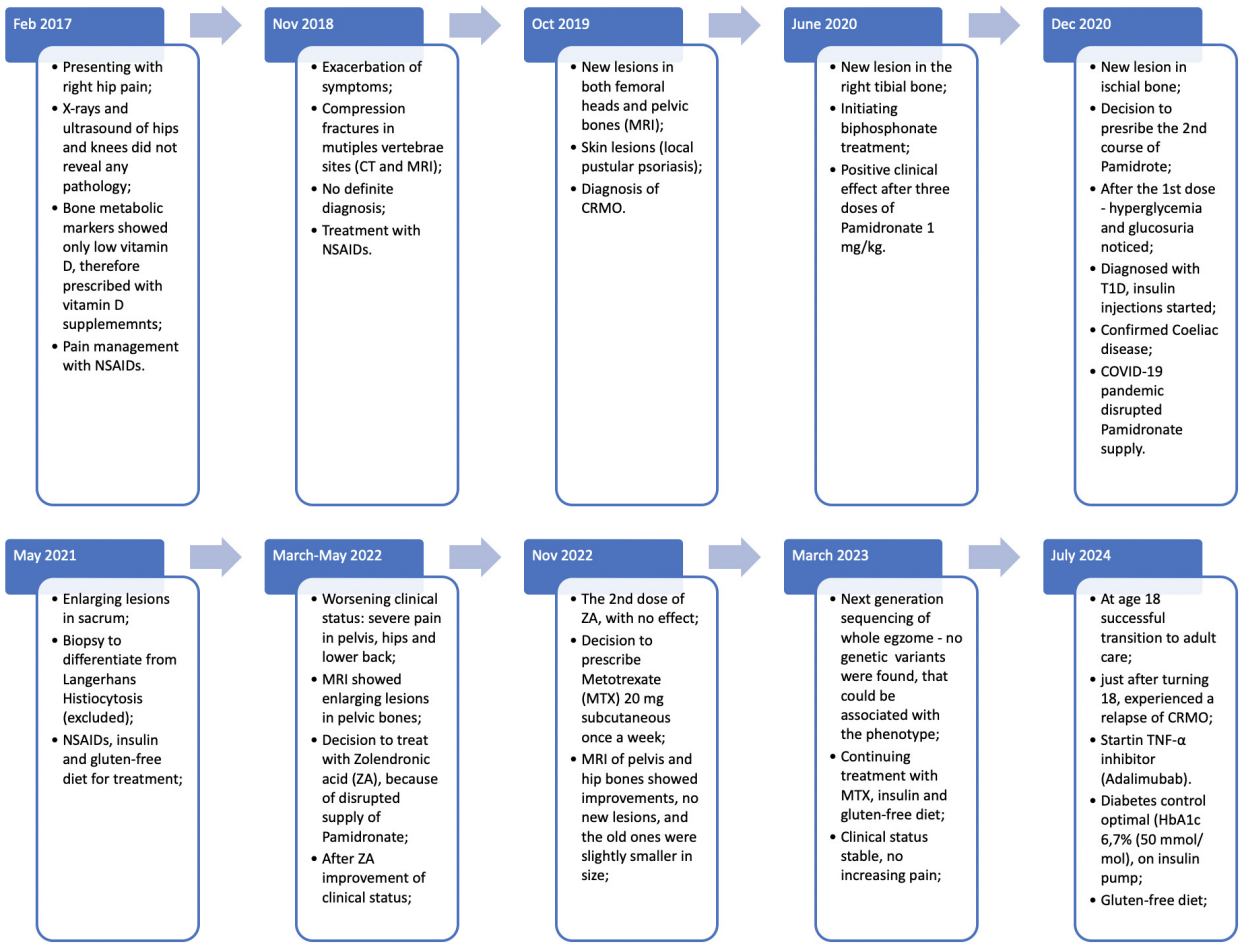
Main timepoints and clinical presentation of the case.

Just after turning 18, in summer of 2024, she had a relapse of CRMO, and experienced a CRMO relapse, prompting the adult rheumatologist to initiate treatment with a TNF-α inhibitor, Adalimumab. Her follow up plan consists of regular visits to rheumatologist and endocrinologist, in order, to track her health alterations and reduce the risk of disease relapse. The main timepoints of clinical course, diagnostics and treatment are presented in **Figure 3**.

## Discussion

We present a unique case of combined autoimmune and autoinflammatory diseases in a young teenager. To our knowledge, this is the first case report of a child with CRMO, T1D and celiac disease. Presently, there is an escalating incidence of CRMO cases concomitant with additional inflammatory or autoimmune conditions such as Crohn’s disease, ulcerative colitis, and skin diseases (4). However, there is a notable absence of documented case reports concerning the coexistence of CRMO and T1D. Consequently, the objective of this case report is to explain shared processes in the etiology and pathogenesis of these disorders.

CRMO sometimes called chronic non-bacterial osteomyelitis (CNO) is an autoinflammatory bone disorder predominantly affecting children and adolescents. Usually typical lesions occur in the metaphysis of long bones, pelvic bones, vertebrae, and other skeletal regions (5). Despite its rarity, with approximately 500 documented cases worldwide, awareness of CRMO is expanding, and the number of reported patients is increasing (6). The clinical presentation lacks specific features; however, pain and swelling in the affected bone regions are the most common (5). In the largest study involving 486 CNO patients from the Eurofever registry, Girschick et al. highlighted diverse clinical manifestations, potentially involving additional inflammatory disorders impacting the skin (psoriasis, acne), digestive system (Crohn’s disease, ulcerative colitis), lymphoid tissue (hepatosplenomegaly, lymphadenopathy), or heart (pericarditis) (7). Typically, CRMO diagnosis is based on the exclusion of other rheumatological diseases. Since 2016 Bristol’s diagnostic criteria have facilitated diagnostics of CRMO. Notably, the authors advocate against bone biopsy in typical pediatric cases (8).

The pathogenesis of CRMO is usually explained by altered functions of innate immune cells. In this autoinflammatory disorder monocytes exhibit a diminished expression of key regulatory cytokines, namely interleukin (IL)-10 and IL-19, which play crucial roles in immune system modulation. Concurrently, there is an upregulation in the production of pro-inflammatory cytokines (IL-1ß, IL-6, tumor necrosis factor (TNF)-α) and various chemokines. The dysregulated cytokine milieu induces osteoclast differentiation and activation, ultimately contributing to the development of bone lesions (3, 6).

In presented case, the definitive diagnosis of CRMO was reached after intensive investigations that took almost 2 years. The progression of clinical symptoms, and multiple lesions with perifocal bone marrow edema in MRI scans guided to the final diagnosis. However, the diagnostic process necessitated the systematic exclusion of alternative etiologies, including malignancy, infection, or other autoimmune conditions. Considering the data available now, we may hypothesize that earlier utilization of MRI for the affected sites or whole-body MRI could bring an accurate diagnosis sooner. Moreover, whole-body MRI is the preferred imaging modality due to its enhanced capacity for lesion detection and identification of asymptomatic lesions in comparison to localized MRI (9).

The second pathology that developed in our patient was a well-known endocrinopathy – T1D. Glycosuria and hyperglycemia were noticed during routine laboratory tests before scheduled pamidronate infusion, and our patient avoided diabetic ketoacidosis. It is known that T1D is caused by the autoimmune destruction of pancreatic beta-cells. There are several known pathways for the activation of apoptosis in beta-cells. Firstly, activated T helper lymphocytes activate macrophages via the release of pro-inflammatory cytokines (IL-1ß, TNF-α, interferon (INF)-γ). Secondly, autoantigen-specific T cytotoxic (CD8) cells are activated, also causing destruction of beta-cells. Moreover, activation of B lymphocytes leads to production of autoantibodies against beta-cell structures (10, 11). Due to complete destruction of beta-cells, individuals become insulin dependent and necessitate lifelong treatment.

After screening for specific markers, following international guidelines for diabetes diagnosis and management (12), patient was diagnosed with the most common diabetes associated autoimmune disorder – celiac disease. Both conditions share identical genetic susceptibility markers, specifically the HLA genotypes (HLA DQ2 and HLA DQ8). After the confirmed diagnosis of celiac disease, the patient was recommended to follow gluten-free diet.

**Figure 4:**
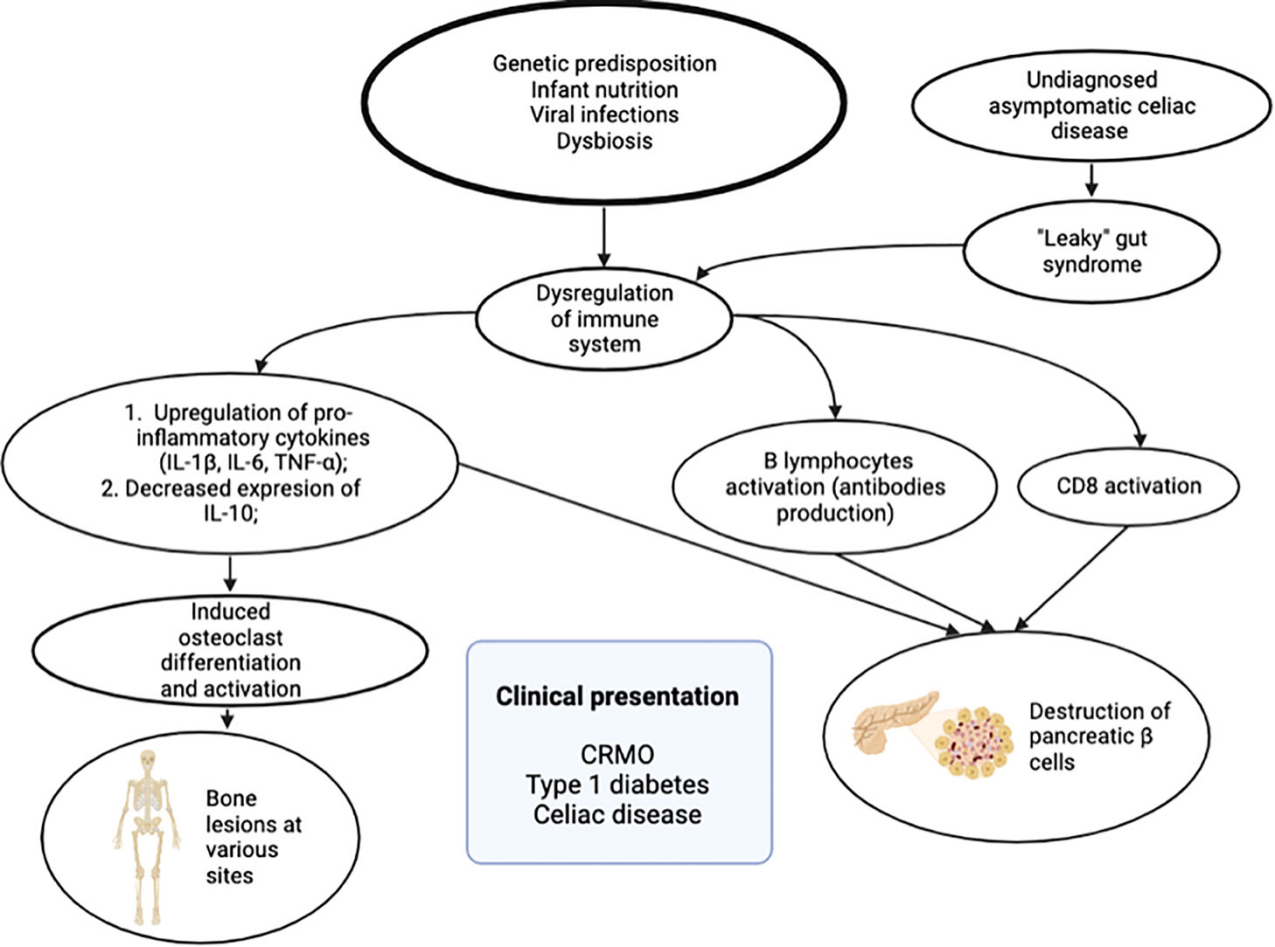
Etiological factors and pathogenesis leading to clinical phenotype: chronic recurrent multifocal osteomyelitis (CRMO), type 1 diabetes, and celiac disease.

Considering all known pathophysiological mechanisms of mentioned disorders, we found out that two possible etiological pathways could lead to this combined pathology. Clearly, this dysregulation of immune system in our patient emerged from interactions between genetic predisposition and various environmental, immunologic factors, like infant nutrition, viral infections, already known to contribute to various immunological disorders (12, 13). First of all, increased released of IL-1ß and TNF-α, as proinflammatory cytokines from macrophages, play an important role in CRMO and T1D pathogenesis. Secondly, we hypothesize that asymptomatic and undiagnosed celiac disease could be the primary disorder in our patient, resulting “leacky gut” syndrome, that is known to predispose the dysregulation of immune system. It is known that due to dysregulation of microbiome and increased intestinal permeability, fragments of gliadin invade the lamina propria, cross the intestinal epithelial barrier, and then they induce immune system response and activation of CD4+ T lymphocytes. The consequence of this is increased levels of pro-inflammatory cytokines, eventually activating B lymphocytes, which differentiate into plasma cells producing autoantibodies (14). Also the activation of CD8 lymphocytes contributes to autoimmune process and destruction of pancreatic insulin producing cells (15). The short scheme of hypothesized etiology and pathogenesis leading to described clinical phenotype is presented in **Figure 4**.

Managing autoimmune or autoinflammatory disease is challenging, especially when dealing with the combination of both these pathologies. In 2018, Childhood Arthritis and Rheumatology Research Alliance (CARRA) provided treatment guidelines for CNO/CRMO (16). NSAIDs are recommended as a first line treatment, however, as monotherapy it is not effective in patients with vertebral lesions, as seen also in our case (6, 16). According to the analysis of reported cases, commonly used treatments were MTX, TNF inhibitors or bisphosphonates (16). Regarding T1D treatment it is clearer: insulin is the only one possible treatment choice (17). For coeliac disease, usually it is enough to be complaint with the gluten-free diet (12).

The main limitation that could be highlighted is the lack of cases of this kind in scientific databases, as we report the unique case to our knowledge. Therefore, we referenced studies discussing the pathophysiology of CRMO and T1D separately, and proposed a potential mechanism that could explain the coexistence of these autoinflammatory and autoimmune disorders. To validate our assumption, we plan to collaborate with a medical biologist to conduct laboratory experiments *in vitro* and animal models. Moreover, it is planned to perform whole genome sequencing, that may help identify genetic variants contributing to this clinical phenotype.

Overall, our case has shown that managing the combination of these disorders was very challenging, with fluctuating clinical course, but finally, we managed to find the most suitable treatment for this individual patient.

## Conclusions

To conclude, as our case report has shown, the combination of autoimmune and autoinflammatory diseases, brings not only a challenging diagnostic process, but also complicated treatment. Therefore, we believe that discussions in multidisciplinary national and international teams could bring precise diagnosis and individualize the treatment quicker for patients with rare or complicated pathologies. Moreover, there is a definite need for the development of new drugs targeting specific pathophysiological mechanisms in autoimmune and autoinflammatory diseases.

## Data Availability

The raw data supporting the conclusions of this article will be made available by the authors, without undue reservation.

## Glossary

ANA: antinuclear antibodies
ANCA: antineutrophil cytoplasmic antibodies
CARRA: Childhood Arthritis and Rheumatology Research Alliance
CBC: complete blood count
CD4: T (helper) lymphocyte
CD8: cytotoxic T cell
CRMO: chronic recurrent multifocal osteomyelitis
CRP: C-reactive protein
CT: computed tomography
DXA: dual energy X-ray absorptiometry
ESR: erythrocyte sedimentation rate
GAD: glutamic acid decarboxylase
GAF3X: antibodies against deaminated gliadin
HbA1c: glycosylated haemoglobin
HLA: human leucocyte antigen
IA-2: tyrosine phosphatase related islet antigen 2
Ig: immunoglobulin
IL: interleukin
INF: interferon
ISPAD: International Society for Pediatric and Adolescent Diabetes
MRI: magnetic resonance imaging
MTX: methotrexate
NGS: next generation sequencing
NSAID: nonsteroidal anti-inflammatory drug
RF: rheumatoid factor
T1D: type 1 diabetes
TB: tuberculosis
TNF: tumor necrosis factor
ZA: zolendronic acid
